# A MgAl-LDH-CuS nanosheet-based thermo-responsive composite hydrogel with nir-responsive angiogenesis inhibitor releasing capability for multimode starvation therapy

**DOI:** 10.1186/s12951-024-02384-w

**Published:** 2024-03-23

**Authors:** Xueyan Liu, Tingting Hu, Yijiang Jia, Shuqing Yang, Yu Yang, Zhuolin Cui, Tao Wang, Ruizheng Liang, Chaoliang Tan, Yuji Wang

**Affiliations:** 1 School of Pharmaceutical Sciences, Capital Medical University, No.10 Xitoutiao, You An Men, Beijing, 100069 P. R. China; 2 Laboratory for Clinical Medicine, Engineering Research Center of Endogenous Prophylactic of Ministry of Education of China, Beijing Laboratory of Biomedical Materials, Beijing, 100069 P. R. China; 3 State Key Laboratory of Chemical Resource Engineering, Beijing Advanced Innovation Center for Soft Matter Science and Engineering, Beijing University of Chemical Technology, Beijing, 100029 P. R. China; 4 Quzhou Institute for Innovation in Resource Chemical Engineering, Quzhou, 324000 P. R. China; 5 Department Electrical and Electronic Engineering, The University of Hong Kong, Pokfulam Road, Hong Kong SAR, 999077 P. R. China

**Keywords:** Layered double hydroxides, Thermo-sensitive hydrogels, Angiogenesis inhibitor, NIR-responsive releases, Starvation therapy

## Abstract

The rapid proliferation of tumors is highly dependent on the nutrition supply of blood vessels. Cutting off the nutrient supply to tumors is an effective strategy for cancer treatment, known as starvation therapy. Although various hydrogel-based biomaterials have been developed for starvation therapy through glucose consumption or intravascular embolization, the limitations of single-mode starvation therapy hinder their therapeutic effects. Herein, we propose a dual-function nutrition deprivation strategy that can block the nutrients delivery through extravascular gelation shrinkage and inhibit neovascularization through angiogenesis inhibitors based on a novel NIR-responsive nanocomposite hydrogel. CuS nanodots-modified MgAl-LDH nanosheets loaded with angiogenesis inhibitor (sorafenib, SOR) are incorporated into the poly(n-isopropylacrylamide) (PNIPAAm) hydrogel by radical polymerization to obtain the composite hydrogel (SOR@LDH-CuS/P). The SOR@LDH-CuS/P hydrogel can deliver hydrophobic SOR with a NIR-responsive release behavior, which could decrease the tumor vascular density and accelerate cancer cells apoptosis. Moreover, the SOR@LDH-CuS/P hydrogel exhibits higher (3.5 times) compressive strength than that of the PNIPAAm, which could squeeze blood vessels through extravascular gelation shrinkage. In vitro and in vivo assays demonstrate that the interruption of nutrient supply by gelation shrinkage and the prevention of angiogenesis by SOR is a promising strategy to inhibit tumor growth for multimode starvation therapy.

**Supplementary Information:**

The online version contains supplementary material available at 10.1186/s12951-024-02384-w.

## Introduction

Malignant tumor is one of the most serious diseases that threaten human health [1–5]. It has been reported that the rapid proliferation of tumors is highly dependent on the oxygen and nutrition supply of tumor blood vessels [6]. Based on this, a theory of starvation therapy was first proposed by Professor Folkman in 1971, which works by blocking the nutrients supply to cancer cells [7]. There are three main strategies for starvation therapy: inhibiting the formation of new blood vessels in tumors through angiogenesis inhibitors, blocking the blood transfusion and oxygen supply function of tumor blood vessels through intravascular embolization, and suppressing the metabolic process of tumor cells by glucose consumption [8–10]. Currently, various biomaterials have been developed for starvation therapy, such as gels [11], magnesium silicide nanoparticles [12] and zeolitic imidazolate framework-8 nanoplatforms [13]. Among them, hydrogels have shown great prospects in starvation therapy due to their excellent biocompatibility, biodegradability and drug delivery ability [14–17]. For example, a composite hydrogel was designed and fabricated to delivery glucose oxidase (GOx), which led to tumor starvation and necrosis through the GOx-mediated nutrition consumption [18]. A nanocomposite hydrogel has been designed to block the blood supply and oxygen supply through extravascular gelation shrinkage, which exhibited a significant inhibitory effect on tumor growth in the short term [19]. However, the strong mutagenicity and adaptability of tumors make hydrogel-mediated single-mode (glucose consumption or intravascular embolization) starvation therapy less effective. In addition, as hydrophilic drug delivery carriers, traditional hydrogels are difficult to achieve efficient loading of hydrophobic angiogenesis inhibitors [20–22]. Even worse, they usually exhibit poor mechanical properties due to the single crosslinking mode [23, 24]. Therefore, it is necessary to develop a new starvation therapy pathway based on the hydrogels that combines the function of inhibiting new blood vessels formation and blocking nutrient supply to tumors.

Tow-dimensional (2D) layered double hydroxides (LDHs) with excellent biodegradability and biocompatibility have been widely explored in biomedical applications, such as cancer therapy, drug delivery, tissue engineering, and imaging diagnosis, etc. [25–29]. Promisingly, recent studies have proved that LDHs can achieve efficient loading of both hydrophilic and hydrophobic drugs [30–36]. As a typical example, Gd&Yb-LDH monolayer nanosheets could serve as hydrophobic drug delivery carriers to achieve the loading of SN38 with an ultrahigh loading rate of 925%, exhibiting extremely high anticancer activity [37]. In addition, as a “molecular container”, MgAl-LDH could improve the bioavailability of Berberine with poor water solubility, which exhibited a hypoglycemic effect [38]. More importantly, LDHs with abundant hydroxyl groups can combine with amide bonds in N-isopropyl acrylamide (NIPAAm, a typical temperature-sensitive monomer) to form hydrogen bonds to prepare temperature-sensitive hydrogels [28, 39, 40]. For example, a novel nanocomposite hydrogel (LDH/PAM) was designed by in situ free radical polymerization, which showed significantly enhanced mechanical properties [41]. Therefore, the construction of LDH-based temperature-sensitive hydrogel is expected to realize the high-efficiency loading of hydrophobic angiogenesis inhibitors and the enhancement of the mechanical properties of hydrogels, eventually achieving a multi-mode starvation therapy combining the inhibition of new blood vessels formation by angiogenesis inhibitors and the blocking of nutrient supply by extravascular gelation shrinkage.

**Fig. 1:**
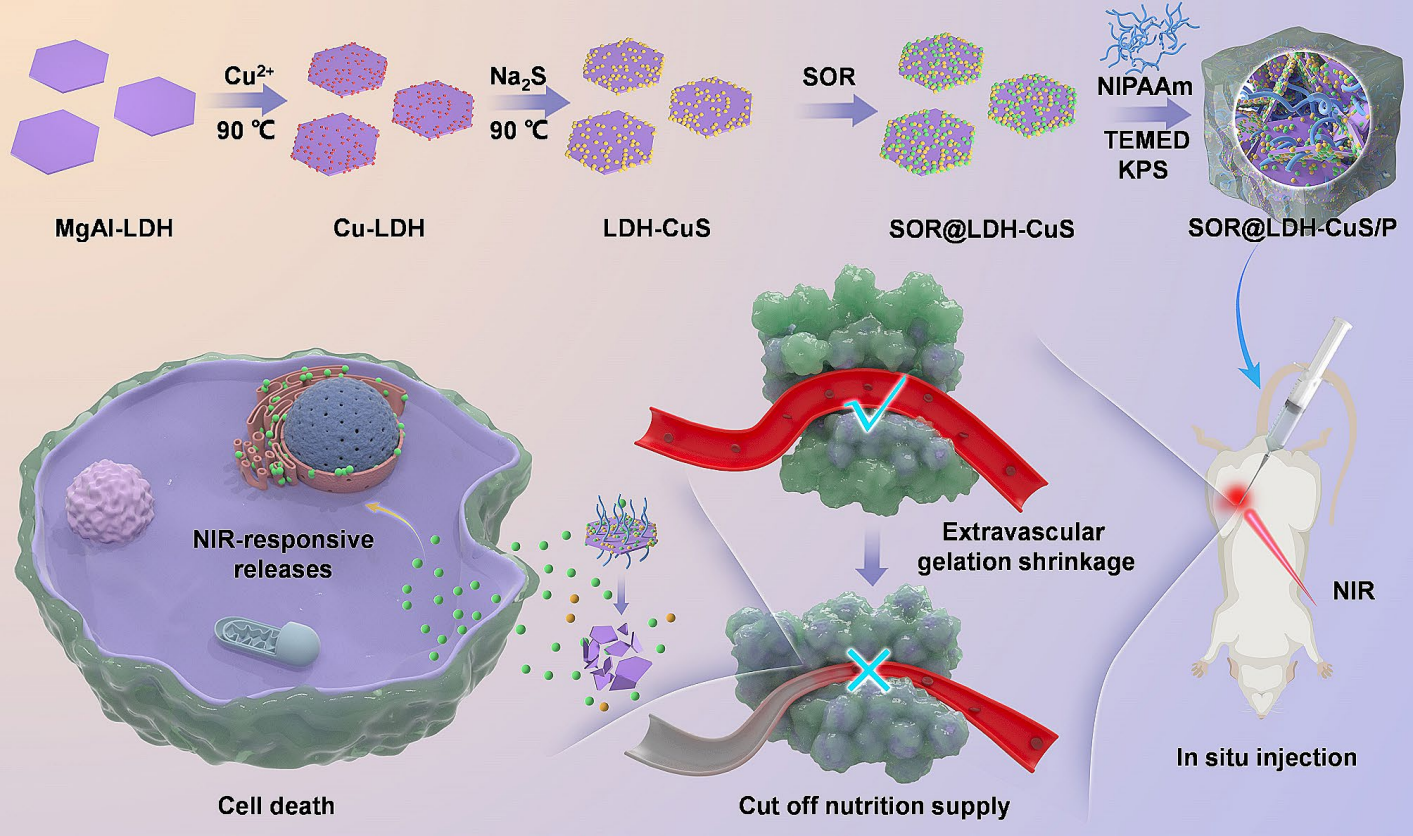
Schematic diagram of the preparation of SOR@LDH-CuS/P hydrogel for multi-mode starvation therapy by blocking the nutrients delivery in tumor through extravascular gelation shrinkage and inhibiting neovascularization through angiogenesis inhibitors

Herein, we design and prepare a multifunctional drug delivery system based on the nanocomposite hydrogels (Fig. 1), and propose a new starvation therapy protocol that could synergistically block the nutrients supply for tumors through extravascular gelation shrinkage and inhibition of neovascularization through sorafenib (SOR, a multi-kinase inhibitor that can inhibit tumor angiogenesis and promote tumor cell apoptosis) [16]. The MgAl-layered double hydroxide (MgAl-LDH) nanosheets are first decorated with CuS nanodots and then loaded with the angiogenesis inhibitor, i.e., SOR, and the prepared composite nanosheets are then incorporated into the poly(n-isopropylacrylamide) (PNIPAAm) hydrogel by a simple radical polymerization to obtain the thermal-responsive composite hydrogel (denoted as SOR@LDH-CuS/P). The SOR@LDH-CuS/P is prepared through in situ free radical polymerization by introducing NIPAAm monomer, N,N,N’,N’-tetramethyl-ethylenediamine (TEMED) accelerator and potassium persulfate (KPS) initiator into LDH-CuS colloid loaded with SOR. The addition of LDH-CuS can not only realize the high efficiency loading and NIR-responsive release of vascular inhibitor SOR, but also enhance the mechanical properties of SOR@LDH-CuS/P hydrogel through the nano-reinforcement effect, whose compressive strength is 3.5 times that of the PNIPAAm hydrogel. More importantly, NIR irradiation could promote the sol-gel transition of SOR@LDH-CuS/P due to the inherent photothermal properties of LDH-CuS nanosheets and the intrinsic phase transition ability of the thermo-responsive PNIPAAm. Furthermore, the results of ex vivo vascular occlusion experiments indicate the considerable potential of this hydrogel in squeezing tumor blood vessels *via* the gelation shrinking. Power doppler imaging and dextran perfusion experiments prove that SOR@LDH-CuS/P irradiated by 1064 nm laser could enhance gel shrinkage, cut off blood and nutrient supply, thereby inhibiting tumor progression. Both in vitro and in vivo tests show that SOR@LDH-CuS/P possesses significant inhibitory effect on cell proliferation and tumor growth for long-acting therapy. All results indicate that SOR@LDH-CuS/P provides a successful paradigm for the development of cancer starvation therapy.

## Results and discussion

### Preparation and characterization of the SOR@LDH-CuS/P hydrogels

**Fig. 2:**
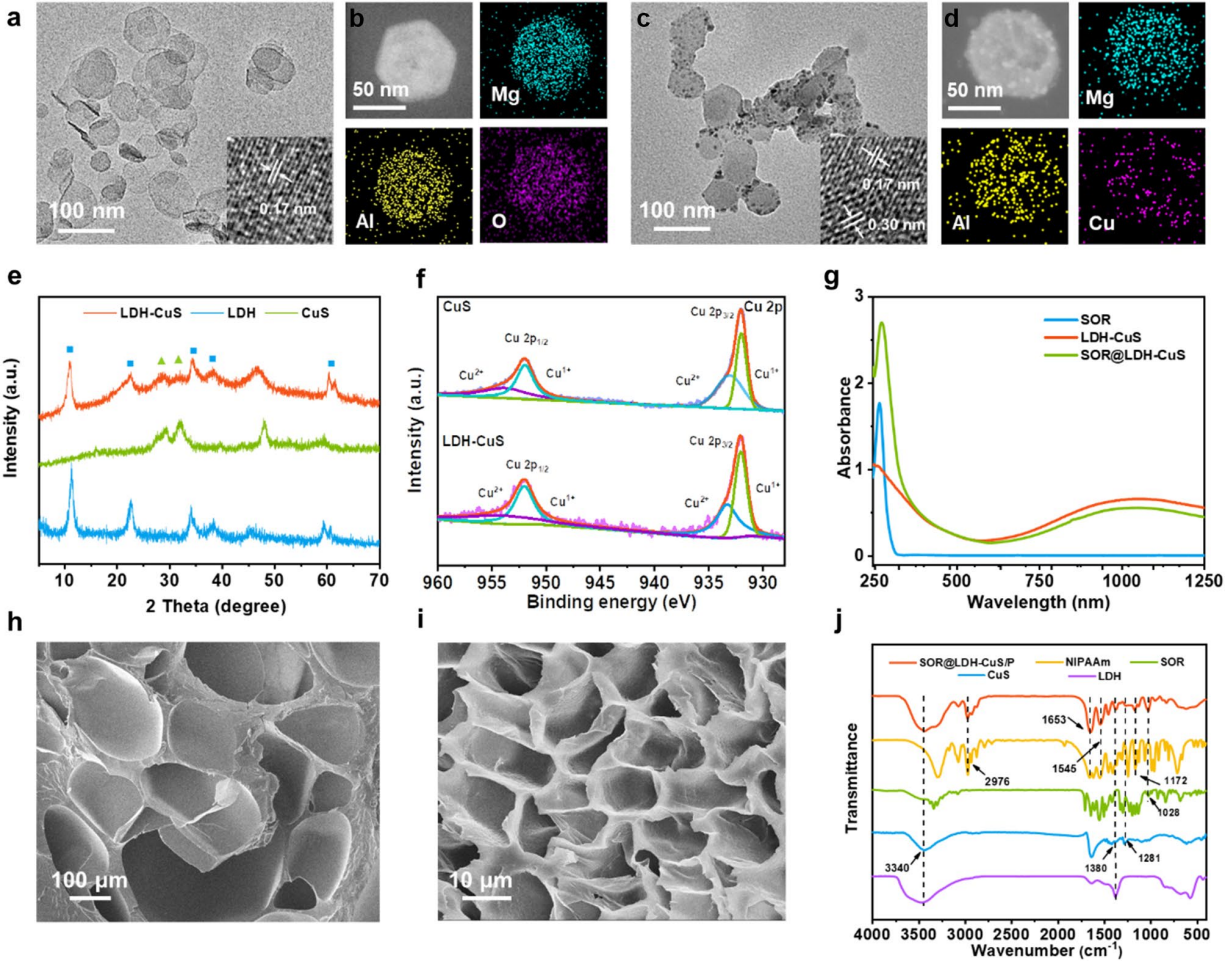
(a) HRTEM images and (b) EDX elemental mapping of MgAl-LDH nanosheets. (c) HRTEM images and (d) EDX elemental mapping of LDH-CuS nanosheets. (e) XRD patterns of MgAl-LDH nanosheets, CuS nanodots, and LDH-CuS nanosheets. (f) XPS Cu 2p spectra of CuS nanodots and LDH-CuS nanosheets. (g) UV-vis-NIR absorption spectra of SOR, LDH-CuS, and SOR@LDH-CuS nanosheets. SEM images of (h) PNIPAAm and (i) SOR@LDH-CuS/P. (j) FT-IR spectra of SOR@LDH-CuS/P, NIPAAm, SOR, CuS, and MgAl-LDH nanosheets

2D MgAl-LDH nanosheets were synthesized through a classical “bottom-up” method according to our previous report [35]. The morphology of MgAl-LDH nanosheets was characterized by high resolution transmission electron microscopy (HRTEM). As shown in Fig. 2a, MgAl-LDH nanosheets display monodispersed hexagonal morphology with a size of 60–100 nm, and the measured lattice fringe spacing of the MgAl-LDH (110) plane is ∼ 0.17 nm, suggesting its good crystallinity. According to the results from dynamic light scattering (DLS), the hydrodynamic diameter of MgAl-LDH nanosheets was 78.82 ± 6.22 nm (Fig. S1), which was consistent with the result of HRTEM. Moreover, the energy dispersive X-ray (EDX) mapping shows the homogeneous distribution of Mg and Al across the LDH nanosheets (Fig. 2b). Then, Cu-doped LDH nanosheets were prepared through an isomorphic substitution method, in which Cu^2+^ partially replaced Mg^2+^ and Al^3+^ [42]. After further sulfidation by Na_2_S, CuS nanodots were obtained in situ on the surface of MgAl-LDH nanosheets to obtain LDH-CuS nanosheets. Inductively coupled plasma mass spectrometry (ICP-MS, Table S1) was used to detect the metal element ratios of MgAl-LDH and LDH-CuS nanosheets, proving that Mg^2+^ and Al^3+^ in the MgAl-LDH were replaced by Cu^2+^ through the isomorphic substitution process. HRTEM image of LDH-CuS nanosheets in Fig. 2c shows that the CuS nanodots were successfully grown in situ on LDH nanosheets with the lattice spacing of 0.30 nm, which was corresponded to the (102) plane of the CuS crystal (Fig. 2c inset). More importantly, the LDH-CuS still maintained a plate-like structure. Meanwhile, the EDX mapping displays that Mg, Al and Cu elements were homogeneously distributed throughout the whole LDH-CuS nanosheets (Fig. 2d). The structural information of LDH-CuS nanosheets was further investigated by XRD (X-ray diffractometry) analysis. In Fig. 2e, typical diffraction peaks of MgAl-LDH nanosheets at 11.24° (003) and 22.66° (006) were observed before and after isomorphic substitution of copper. In addition, typical (102) and (103) diffraction peaks at 2*θ* = 29.28° and 31.99° in LDH-CuS nanosheets matched well with the CuS nanodots, verifying their crystal structure. X-ray photoelectron spectroscopy (XPS) was also utilized to analyze the valence state of Cu in LDH-CuS nanosheets (Fig. S2). Figure 2f shows that the characteristic peaks of Cu 2p at 952.08, 932.03, 954.13, and 933.02 eV can be attributed to Cu^1+^ 2p_1/2_, Cu^1+^ 2p_3/2_, Cu^2+^ 2p_1/2_, Cu^2+^ 2p_3/2_, respectively, indicating the coexistence of Cu^1+^ and Cu^2+^ in the LDH-CuS nanosheets, which is consistent with free CuS nanodots [42]. This result confirmed the successful growth of CuS nanodots on LDH nanosheets.

Based on the superior drug delivery ability of 2D LDH nanosheets, LDH-CuS nanosheets were used as a drug carrier to achieve the loading of SOR. The UV-vis-NIR absorption spectrum of SOR@LDH-CuS nanosheets exhibits the absorption peak of LDH-CuS at ∼ 1100 nm and the characteristic peak of SOR at 265 nm (Fig. 2g) [43], indicating the successful loading of SOR molecules onto LDH-CuS nanosheets. Their combination was further confirmed by Zeta potential. In Fig. S3, the zeta potentials of SOR, MgAl-LDH, and CuS nanodots are 12.7, 27.5 and − 13.9 mV, respectively. Therefore, SOR is just loaded on the surface of LDH-CuS nanosheets *via* the hydrogen bond between MgAl-LDH and SOR and the electrostatic attraction between CuS and SOR. The loading performance with various mass ratios of LDH-CuS: SOR was investigated by UV-vis absorption spectroscopy. In Fig. S4, with the decrease of LDH-CuS: SOR mass ratio, the load capacity (LC) increased slightly but the entrapment efficiency (EE) decreased obviously. Considering both LC and EE, the final mass ratio of LDH-CuS:SOR was determined to be 1:2 with the LC and EE of 184.5% and 92.3%, respectively.

Subsequently, SOR@LDH-CuS/P nanocomposite hydrogels were prepared through in situ free radical polymerization by introducing NIPAAm monomer, KPS initiator and TEMED accelerator into SOR@LDH-CuS. For comparison, the conventional chemically crosslinked hydrogels (abbreviated as PNIPAAm) using N,N’-methylenebis(acrylamide) (BIS) as the crosslinker were prepared [44, 45]. As shown in Fig. 2h and i, the pore size of the SOR@LDH-CuS/P hydrogel was reduced compared with PNIPAAm, suggesting that LDH-CuS nanosheets have the potential to enhance the mechanical strength of composite hydrogels by augmenting cross-linking joints and weaving 3D spatial network [19]. The chemical composition of SOR@LDH-CuS/P hydrogels was studied *via* Fourier transform infrared (FT-IR) spectroscopy. In Fig. 2j, the bands at 1653 cm^− 1^, 1545 cm^− 1^ (stretching vibration of amide) for NIPAAm, 1380 cm^− 1^ (symmetric stretching vibration of N-O) for LDH-CuS and 1028 cm^− 1^ (stretching vibration of C-O-C) for SOR were observed in the SOR@LDH-CuS/P sample, demonstrating the successful preparation of SOR@LDH-CuS/P hydrogel.

### Rheology and photothermal performance of the SOR@LDH-CuS/P hydrogels

**Fig. 3:**
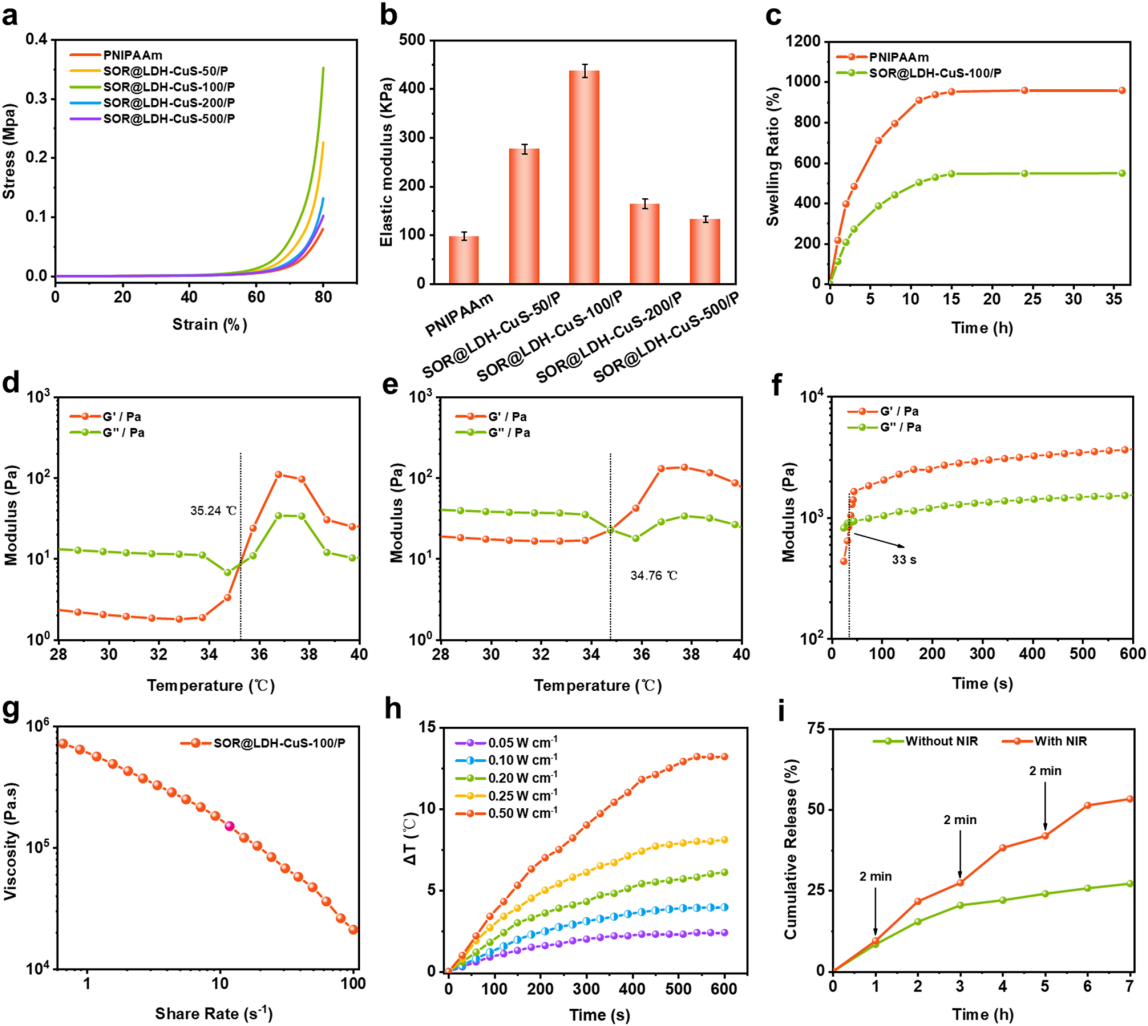
(a) Compressive stress–strain curves of PNIPAAm, SOR@LDH-CuS-50/P, SOR@LDH-CuS-100/P, SOR@LDH-CuS-200/P, and SOR@LDH-CuS-500/P, respectively. (b) Elastic modulus of PNIPAAm, SOR@LDH-CuS-50/P, SOR@LDH-CuS-100/P, SOR@LDH-CuS-200/P, and SOR@LDH-CuS-500/P, respectively, determined as strain = 80%. (c) Swelling Ratio of PNIPAAm and SOR@LDH-CuS-100/P. Temperature-dependent rheological curves of (d) PNIPAAm and (e) SOR@LDH-CuS-100/P. (f) Time-dependent rheological analysis of SOR@LDH-CuS-100/P performed at 25 °C and 1% of strain. (g) The flow sweep of SOR@LDH-CuS-100/P at 25 °C. (h) Temperature profiles of SOR@LDH-CuS-100/P under 1064 nm laser irradiation at various power densities (0.05, 0.10, 0.20, 0.25 and 0.50 W cm^− 2^). (i) The release behaviors of SOR from SOR@LDH-CuS-100/P with or without NIR irradiation

Based on the aforementioned results, various dynamic measurements were carried out to analyze the mechanical properties of SOR@LDH-CuS/P nanocomposite hydrogels. Compressive stress-strain inspection was used to quantitatively investigate the compressive strength of SOR@LDH-CuS/P hydrogels containing various concentrations (50, 100, 200 and 500 µg mL^− 1^) of LDH-CuS (denoted as SOR@LDH-CuS-50/P, SOR@LDH-CuS-100/P, SOR@LDH-CuS-200/P, and SOR@LDH-CuS-500/P). As shown in Fig. 3a, all SOR@LDH-CuS/P hydrogels displayed a more significant anti-compression ability compared with PNIPAAm, proving that the multiple noncovalent interactions (hydrogen bond) between LDH-CuS and NIPAAm substantially increased the mechanical properties of SOR@LDH-CuS/P hydrogels (Fig. 3a,b). Among them, SOR@LDH-CuS-100/P exhibited much higher (3.5 times) compressive strength than that of PNIPAAm, proving that the introduction of LDH-CuS imparted SOR@LDH-CuS/P hydrogels with larger elastic modulus arising from increased mechanical properties. The above results suggest the considerable potential of SOR@LDH-CuS/P in squeezing tumor blood vessels through extravascular gelation shrinkage. Since cross-linking density can significantly affect the swelling behavior of hydrogels, the swelling ratio of SOR@LDH-CuS-100/P and PNIPAAm hydrogel was evaluated. Compared with PNIPAAm, the swelling ratio of SOR@LDH-CuS-100/P was significantly decreased, which further proved that the increase of hydrogel cross-linking density led to the enhancement of mechanical properties (Fig. 3c). Moreover, temperature sweep was determined to demonstrate the temperature-triggered gelation of different hydrogel samples. Figure 3d,e showed that both PNIPAAm and SOR@LDH-CuS-100/P exhibited temperature-responsive reversible sol-gel transition, and the lower critical solution temperature (LCST) was detected at around 35 °C. Such a suitable gelation temperature and efficient strength are beneficial for in vivo implantation. The sol-gel transition of SOR@LDH-CuS-100/P was detected by inverted test tube method. As shown in Fig. S5a, the SOR@LDH-CuS-100/P exhibited good liquidity at 25 °C and rapid gelation at 37 °C. Subsequently, time sweep rheological analysis in Fig. 3f showed that the SOR@LDH-CuS-100/P could gel within 33 s at 37 °C. In addition, the injectability of SOR@LDH-CuS-100/P was tested by extruding it through a syringe. As shown in Fig. S5b, the letter “Gel” was successfully written using this hydrogel. Moreover, the shear thinning properties of SOR@LDH-CuS-100/P were also investigated by shear flow sweep assay. It was found that the viscosity of SOR@LDH-CuS-100/P decreased steadily with increasing shear rate from 1 to 100 s^− 1^ (Fig. 3g), further confirming the good injectability of the SOR@LDH-CuS-100/P hydrogel.

It is generally known that NIR light (700–1350 nm) is the best ‘biological optical window’ with strong tissue penetration [46–48]. Motivated by the significant absorbance of LDH-CuS in the NIR-II region (Fig. 2g), we tested its temperature change under 1064 nm laser irradiation at various power densities. As shown in Fig. 3h and Fig. S6, both LDH-CuS and SOR@LDH-CuS-100/P resulted in obvious temperature changes at low power densities, proving the excellent photothermal performance of SOR@LDH-CuS-100/P, which was conducive to the rapid sol-gel transition and the promotion of gel shrinkage. In addition, the release behavior of SOR under NIR irradiation was further investigated. As expected, the cumulative release of SOR from SOR@LDH-CuS-100/P reached 53.3% after 3 cycles of ON/OFF laser irradiation, much higher than that of SOR@LDH-CuS-100/P without NIR irradiation (27.1%) (Fig. 3i). This result confirmed the NIR-responsive SOR release, which is beneficial for remotely controlled drug delivery.

### Synergistic antitumor effect in vitro.

**Fig. 4:**
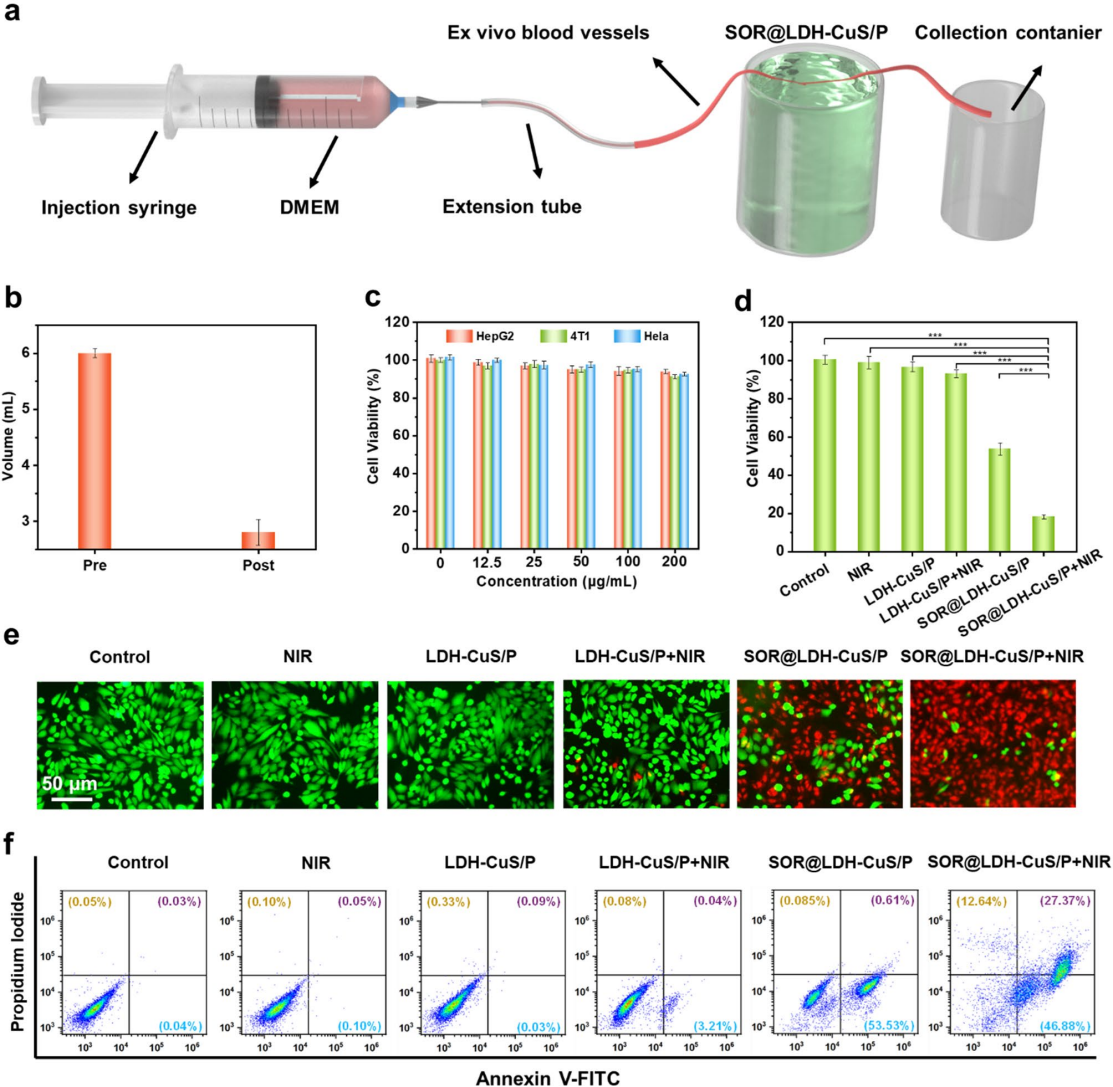
(a) Schematic illustration of experimental apparatus for ex vivo vascular occlusion test. (b) DMEM volume passing the ex vivo blood vessels before (left) and after (right) gelation of SOR@LDH-CuS/P. (c) MTT results of 4T1, Hela and HepG2 cells incubated with LDH-CuS/P hydrogels containing various concentrations of LDH-CuS. (d) Cell viability of HepG2 cells after different treatments: (1) control, (2) NIR, (3) LDH-CuS/P, (4) LDH-CuS/P + NIR, (5) SOR@LDH-CuS/P, (6) SOR@LDH-CuS/P + NIR. (e) Calcein-AM/PI staining images and (f) apoptosis analysis by flow cytometry after different treatments

As previously demonstrated, LDH-CuS significantly improved the rheological and mechanical properties of SOR@LDH-CuS/P hydrogels. Therefore, SOR@LDH-CuS-100/P (if there is no special indication, SOR@LDH-CuS/P refers to SOR@LDH-CuS-100/P) is expected to occlude oxygen and nutrition supply by squeezing tumor blood vessels through the internal stress caused by extravascular gelation shrinkage. To verify this, we used ex vivo blood vessels to explore how this gelation shrinkage occludes blood flow [19]. As shown in Fig. 4a, high glucose Dulbecco’s modified eagle medium (DMEM) was used to simulate blood and the flow rate was set to 72 mL h^− 1^, which is approximately identical to the actual flow rate of blood in rats. It was found that the flow volume of DMEM was collected to be 6 mL in 5 min before gelation, while the flow volume of DMEM sharply decreased to 2.8 mL after gelation (Fig. 4b), indicating that the shrinking of SOR@LDH-CuS/P hydrogels could effectively block blood flow and reduce nutrition supply.

The biocompatibility of nanomaterials is essential for biomedical applications [25]. We prepared LDH-CuS/PNIPAAm (LDH-CuS/P) hydrogel by in situ free radical polymerization without SOR. To evaluate the biosafety of LDH-CuS/P hydrogel, three kinds of cells (4T1, Hela, HepG2) were incubated with LDH-CuS/P hydrogel, and the cell viability was determined by methyl thiazolyl tetrazolium (MTT) assay. The results in Fig. 4c showed that LDH-CuS/P hydrogel exhibited negligible cytotoxicity on 4T1, Hela and HepG2 cells with different concentrations from 12.5 to 200 µg mL^− 1^ (concentration of LDH-CuS in LDH-CuS/P), demonstrating its excellent biocompatibility. The cytotoxicity of SOR@LDH-CuS/P hydrogel was also evaluated with HepG2. As shown in Fig. 4d, individual NIR irradiation and LDH-CuS/P hydrogel showed unobvious cytotoxicity to HepG2 cells. However, after incubation with SOR@LDH-CuS/P hydrogel, the cell viabilities of HepG2 cells obviously declined owing to the release of SOR. Moreover, HepG2 cells exhibited significantly enhanced damage when incubated with SOR@LDH-CuS/P for 24 h accompanied by NIR irradiation (6 min, 0.5 W cm^− 2^), which could be attributed to the promotion of SOR release by NIR irradiation. Calcein acetoxymethyl ester and propidium iodide (Calcein-AM/PI) staining was further applied to visualize the distribution of viable and dead cells (Fig. 4e). It was found that the red fluorescence (represents the dead cells) in SOR@LDH-CuS/P + NIR irradiation group was significantly enhanced compared with other groups, further demonstrating the excellent cancer cell killing ability of thermosensitive hydrogels (SOR@LDH-CuS/P). Similar results were also obtained using flow cytometry assay (Fig. 4f), in which 86.9% apoptosis was observed in HepG2 cells treated with SOR@LDH-CuS/P + NIR group, confirming the effective anticancer performance of SOR@LDH-CuS/P hydrogel under NIR irradiation.

### Synergistic antitumor effect in vivo.

**Fig. 5:**
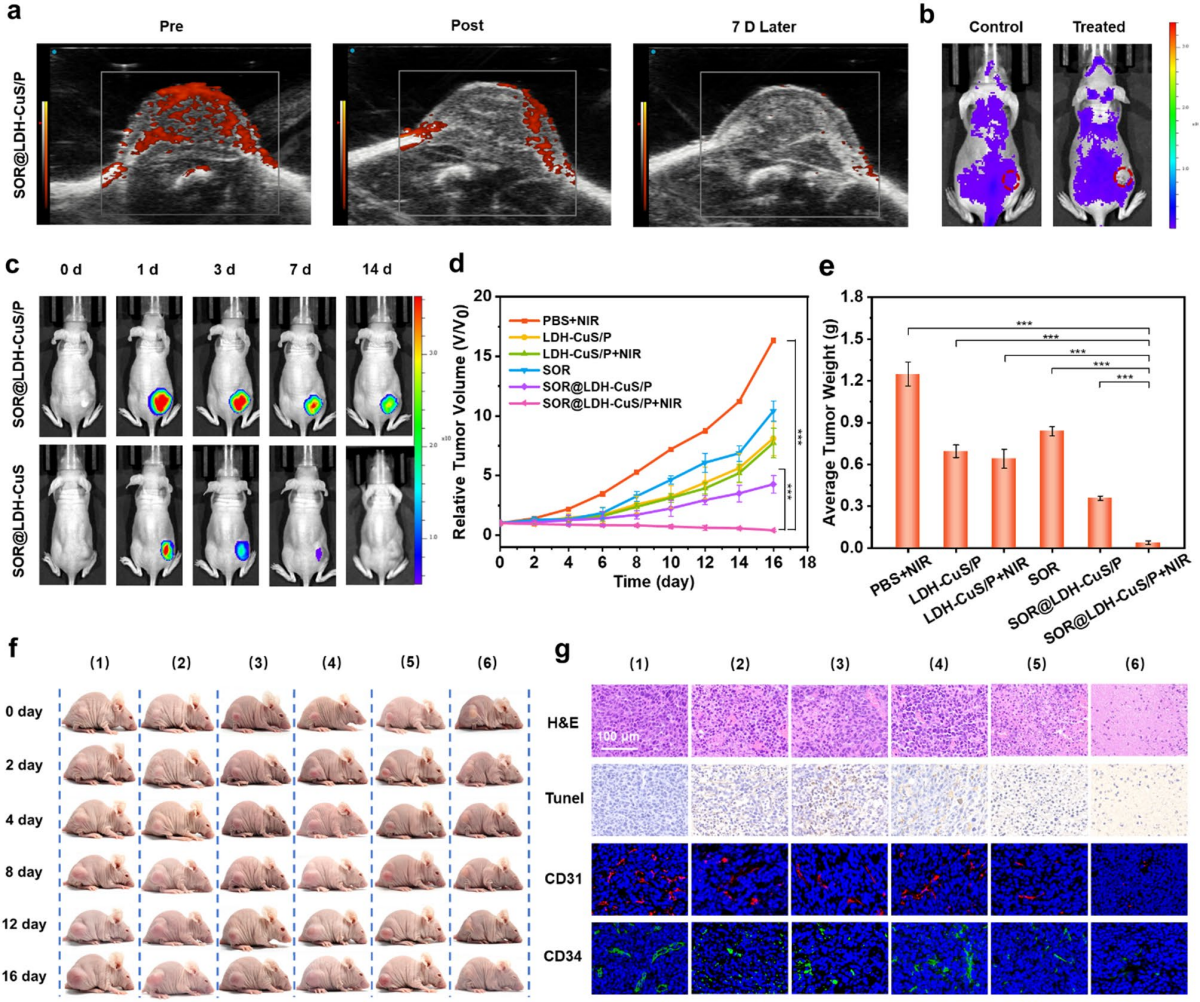
(a) PDI images of HepG2 tumors at different time points, pre-, post- and 7 day post-treatment with SOR@LDH-CuS/P. (b) Fluorescence images of mice after injection of FITC-labeled dextran. (c) Fluorescence images of mice after injection of SOR@LDH-CuS/P and SOR@LDH-CuS modified with Cy7 at various time points. (d) Tumor growth tendency, (e) corresponding average tumor weight and (f) digital photographs of mice with different treatments: (1) PBS + NIR, (2) LDH-CuS/P, (3) LDH-CuS/P + NIR, (4) SOR, (5) SOR@LDH-CuS/P, (6) SOR@LDH-CuS/P + NIR. Data are presented as mean values ± s.d. (*n* = 6). ***p < 0.001. (g) H&E, TUNEL, CD31 and CD34 staining assays of tumor tissue slices from different groups of mice after 16-day treatment

Encouraged by the above exciting results in vitro, the in vivo anticancer performance of SOR@LDH-CuS/P hydrogel was further evaluated using HepG2 tumor-bearing mice. To confirm that extravascular gelation shrinkage could cause vascular narrowing and block blood & nutrition supply, power doppler imaging (PDI) was utilized to assess blood flow distribution in tumors [19, 49]. As shown in Fig. 5a, blood flow signal of mice treated with SOR@LDH-CuS/P hydrogel was significantly weakened compared with the mice before injection. The occluded blood supply remained unrecovered even after 7 days, indicating that the shrinking of the hydrogels could effectively block tumor vessels. To determine whether the decrease of nutrition supply in tumor is responsible for the occluded blood supply caused by gelation shrinkage, in vivo perfusion study was performed using FITC-labeled dextran. In Fig. 5b, the fluorescence intensity of tumor in SOR@LDH-CuS/P hydrogel-treated mice was obviously weaker than that in the control group, revealing that dextran could not enter the tumor because of the vascular occlusion. These findings proved that vascular occlusion in tumors led to the blood flow drop and nutrient supply occlusion. In addition, the in vivo residence of SOR@LDH-CuS/P was evaluated by fluorescence imaging technique [50]. It can be seen from Fig. 5c and S7 that the fluorescence signal at the tumor site in SOR@LDH-CuS/P hydrogel group was much stronger than that in SOR@LDH-CuS group at the same time point, proving the longer retention time of SOR@LDH-CuS/P than that of SOR@LDH-CuS in tumors. Considering that SOR@LDH-CuS could cause temperature change due to the photothermal properties of LDH-CuS nanosheets, the temperature of the tumors of mice after treatment with SOR@LDH-CuS/P plus 1064 laser irradiation was monitored using an infrared thermal camera. In Fig. S8, compared with the alone NIR group with a temperature of 37.6 °C after total 6 min of irradiation, the tumor site temperature of the mice treated with SOR@LDH-CuS/P + NIR reached 41.0 ℃. Such a temperature cannot induce the photothermal therapeutic effect, but is conducive to promoting the sol-gel transition of SOR@LDH-CuS/P and the release of SOR.

After that, in vivo antitumor studies were conducted to evaluate the potential of SOR@LDH-CuS/P for multi-mode starvation therapy. Balb/c nude mice bearing HepG2 tumors were randomly divided into six groups when the tumors reached 80 mm^3^: (1) PBS + NIR, (2) LDH-CuS/P, (3) LDH-CuS/P + NIR, (4) SOR, (5) SOR@LDH-CuS/P, and (6) SOR@LDH-CuS/P + NIR. The tumor volume of mice in different treatment groups was monitored within 16 days. As shown in Fig. 5d and S9, the PBS group with NIR irradiation exhibited significant tumor growth, while the tumor growth of LDH-CuS/P and LDH-CuS/P + NIR groups was obviously inhibited with a tumor growth inhibition rate (TGI) of 50.3% and 52.7%, respectively, suggesting that the shrinking of the hydrogels in vivo could effectively block tumor vessels and then inhibit tumor growth. It should be noted that compared with LDH-CuS/P hydrogel group, the tumor growth of LDH-CuS/P + NIR group was slightly slower, which may be related to the photothermal effect of LDH-CuS/P to promote gel shrinkage. The tumor growth of SOR@LDH-CuS/P group was significantly slower than that of free SOR group with a TGI increased from 36.3 to 73.9%, attributing to the synergistic effect of hydrogel shrinking and sustained release of SOR from hydrogel. The most significant tumor growth inhibition was found in the SOR@LDH-CuS/P + NIR group with a TGI up to 97.6%, indicating the excellent multi-mode therapeutic effect due to the photothermal-promoted SOR release and vasoconstriction arising from the shrinking of SOR@LDH-CuS/P hydrogel under NIR irradiation. The tumors weight (Fig. 5e) and digital photos of mice (Fig. 5f) showed the same trend as the tumor growth curves, proving the excellent antitumor effect of SOR@LDH-CuS/P under NIR irradiation. Subsequently, hematoxylin and eosin (H&E) and TdT-mediated dUTP nick end labeling (TUNEL) staining assays toward tumor slices were used to investigate the cell proliferation and apoptosis in tumor tissue (Fig. 5g) [51]. H&E staining revealed the best tumor killing effect of SOR@LDH-CuS/P + NIR group because of the obvious cytosol degradation and nuclear shrinkage found in tumor tissue. TUNEL staining further verified the anticancer performance of SOR@LDH-CuS/P under NIR irradiation. As the typical markers of vascular endothelial cells, CD31 and CD34 were used to investigate the inhibition degree of angiogenesis [11, 19]. In Fig. 5g, a decrease in blood vessel density was observed in SOR@LDH-CuS/P + NIR group, indicating that SOR@LDH-CuS/P hydrogel could effectively prevent angiogenesis at the tumor site and inhibit tumor growth. In summary, all the results demonstrated that SOR@LDH-CuS/P with NIR irradiation could not only reduce blood vessel proliferation, but also induce a high proportion of tumor necrosis, eventually achieving the high-efficiency tumor starvation therapy.

In addition, there was no significant change in the body weight of each group of mice during 16-day treatment (Fig. S10). H&E staining images of different organs in mice treated with PBS and SOR@LDH-CuS/P showed no noticeable pathological abnormalities or irregularities (Fig. S11). Besides, in vivo toxicity of SOR@LDH-CuS/P was also assessed through blood biochemical analysis (Fig. S12). The results showed that there were no obvious changes of blood cell counts as well as kidney and liver function markers for the control and SOR@LDH-CuS/P hydrogel groups, demonstrating the high biosafety of SOR@LDH-CuS/P. More importantly, the survival time of HepG2 tumor-bearing mice treated with SOR@LDH-CuS/P + NIR was significantly prolonged compared with other groups (Fig. S13).

## Conclusion

In summary, we successfully designed a novel NIR-responsive injectable nanocomposite hydrogel (SOR@LDH-CuS/P) through in situ radical polymerization by introducing NIPAAm monomer, KPS initiator and TEMED accelerator into SOR@LDH-CuS, which could achieve efficient cancer therapy by blocking the nutrients delivery in tumor through extravascular gelation shrinkage and inhibiting neovascularization through angiogenesis inhibitors SOR. The addition of LDH-CuS significantly enhanced the compressive strength of SOR@LDH-CuS/P and endowed it with NIR-responsive ability, which could promote the extravascular gelation shrinkage and SOR release. Compared with PNIPAAm, the compressive strength of SOR@LDH-CuS/P enhanced obviously (3.5 times). In addition, the cumulative release of SOR from SOR@LDH-CuS/P reached 53.3% under NIR irradiation, much higher than that without NIR irradiation (27.1%). Importantly, both PDI images and perfusion experiment with dextran tests proved that the shrinking of SOR@LDH-CuS/P could effectively block mainstream/peripheral vessels and decrease blood & nutrition supply. Therefore, this research provides a new smart drug delivery system based on thermosensitive hydrogel for multi-mode starvation therapy, which exhibited excellent drug delivery and antitumor activity, showing great potential for the local and long-term treatment of malignant tumor.

## Materials and methods

### Materials

Sodium hydroxide (NaOH), sodium nitrate (NaNO_3_), aluminium nitrate (Al(NO_3_)_3_·9H_2_O), magnesium nitrate (Mg(NO_3_)_2_·6H_2_O), formamide, KPS, MTT, and SOR were purchased from Aladdin Chemical. Co. Ltd (shanghai, China). Cupric chloride (CuCl_2_·2H_2_O) was obtained from Fuchen Chemical Reagents Factory (Tianjin, China). N,N,N’,N’-tetramethylethylenediamine (TEMED), polyvinyl pyrrolidone (PVP) and N-isopropylacrylamide (NIPAAm) were purchased from Macklin Biochemical Co. Ltd (shanghai, China). NIPAAm was further purified with n-hexane by re-crystallization prior to use. Fetal bovine serum (FBS) was acquired from Excell Bio. Co., Ltd. (Shanghai, China). Penicillin/streptomycin, 0.25% trypsin-EDTA and DMEM were purchased from Gibco (Invitrogen, Carlsbad, CA). Phosphate buffered saline (PBS) and living/dead cell double-staining kit (Calcein-AM/PI) were bought from Solarbio Science & Technology Co, Ltd (Beijing, China). Annexin V-FITC & propidium iodide apoptosis detection kit and BIS were obtained from Sigma-Aldrich Company (St. Louis, MO, USA). All reagents (except NIPAAm) were of analytical purity and used without further modification.

### Characterizations

Powder X-ray diffraction (XRD) patterns were performed on a Shimadzu XRD-6000 diffractometer equipped with a Cu Kα radiation source (λ = 0.15406 nm). HRTEM images were captured by the JEM-2010 transmission electron microscope (JEOL, Tokyo, Japan) with an accelerated voltage of 200 kV. XPS was obtained using an X-ray photoelectron spectrometer with Al Kα X-ray source (Escalab 250Xi, Thermo Scientific, USA). Hydrodynamic sizes and zeta potentials were determined by DLS (Zeta sizer Nano ZS, Malvern Instruments, U.K.). FT-IR spectra of the samples were measured from 4000 to 400 cm^− 1^ on a Vector 22 (Bruker) at room temperature with a resolution of 2 cm^− 1^. The metal element ratios of the samples were detected by ICP-MS. The freeze-dried hydrogels were sputter-coated with gold (2 nm thickness, MC1000, Hitachi Ltd., Japan), and then visualized using a field-emission scanning electronic microscope (FESEM, Zeiss SUPRA 55, Germany) with an accelerating voltage of 20 kV. The swelling characteristics of hydrogels were determined by the equilibrium swelling index. Briefly, dried hydrogels disks were weighed (Wdry), then immersed in PBS (pH = 7.4) and shaken on a horizontal shaker at 100 rpm until reaching the swelling equilibrium state. Next, the hydrogels were removed from the solution, the free liquid was sucked away with filter paper, and the weight was recorded as W_swollen_. The swelling ratio of different hydrogels was quantified with equation: Swelling ratio = (W_swollen_-W_dry_)/W_dry_*100%. In vitro cell apoptosis analysis was conducted on Flow cytometer (MoFlo XDP, Beckman Coulter). All cellular fluorescence images were performed on a Leica confocal laser scanning microscope (Leica DM6000M, Germany). Serum biochemistry analysis was conducted on an automated hematology analyzer (Bayer Advia 2120), and the blood cell counts were performed on an automatic biochemical analyzer (Olympus AU400).

### Synthesis of MgAl-LDH nanosheets

The preparation of MgAl-LDH nanosheets was according to a previously reported bottom-up method [35]. Firstly, solution A: Mg(NO_3_)_2_·6H_2_O (0.0008 mol) and Al(NO_3_)_3_·9H_2_O (0.0004 mol) were dissolved in 20 mL of ultrapure water. Solution B: NaOH (0.006 mol) was dissolved in 20 mL of ultrapure water. Solution C: NaNO_3_ (0.0002 mol) was dissolved in 15 mL of ultrapure water and then mixed with formamide (5 mL). Subsequently, solution A and B were simultaneously dropped into solution C at 90 ℃ and stirred for 20 min. Then, the obtained product was collected after washing with ethanol and ultrapure water three times through centrifugation. Finally, to remove the excessive formamide, the precipitation was treated with dialysis (8 kDa) for 24 h.

### Synthesis of LDH-CuS nanosheets

In general, the LDH-CuS nanosheets were synthesized through an isomorphic substitution method, in which Cu^2+^ replaced Mg^2+^ and Al^3+^ in MgAl-LDH, and the seeds were grown to CuS nanodots on MgAl-LDH nanosheets [41]. Briefly, 10 mg of MgAl-LDH nanosheets, 10 mg of CuCl_2_ and 400 mg of PVP were added in 10 mL of ultrapure water. Then, the mixture was stirred in an oil bath at 90 °C for 10 min. Subsequently, 120 µL Na_2_S solution (1 mol L^− 1^) was added to the above mixture and stirred at 90 °C for another 20 min. Finally, the LDH-CuS nanosheets were collected and washed with ultrapure water three times.

### Synthesis of SOR@LDH-CuS nanosheets

20 mg of SOR was first dissolved in 1 mL of dimethyl sulfoxide (DMSO). To prepare SOR@LDH-CuS with different mass ratios (LDH-CuS: SOR = 2: 1, 1:1, 1:2, 1: 4), different volumes of SOR DMSO solution were added into LDH-CuS (concentration of LDH-CuS: 5 mg mL^− 1^) suspension and stirred for 24 h. Then, the excessive DMSO and SOR were removed through centrifugation at 7000 r/min three times. Finally, the obtained SOR@LDH-CuS nanosheets were re-dispersed in ultrapure water.

### Synthesis of composite hydrogels

The SOR@LDH-CuS/P composite hydrogels were prepared by free radical polymerization. Briefly, different concentrations of SOR@LDH-CuS (50, 100, 200 and 500 µg mL^− 1^) and monomer NIPAAm (2 g) were dispersed in 10 mL ultrapure water. Then the mixture was purged with nitrogen gas for at least 0.5 h to remove oxygen. After that, the initiator KPS (0.02 g) and the accelerator TEMED (20 µL) were added at 0 ice-water bath, followed by stirring for 30 min to yield a homogeneous dispersion. In addition, the PNIPAAm hydrogels crosslinked by BIS were prepared for comparison in the same way. Finally, the unreacted monomer was removed by a dialysis (8 kDa) treatment. The obtained SOR@LDH-CuS/P hydrogels were placed at 4 °C.

### The determination of LC and EE

The concentration of SOR in the initial solution and supernatant was detected using UV-vis spectroscopy based on the characteristic absorption peak of SOR at 267 nm. The LC and EE of SOR were calculated as follows:

LC = (W_Fed_-W_Non−encapsulated_)/W_LDH−CuS_ *100%.

EE = (W_Fed_-W_Non−encapsulated_)/W_Fed_ *100%.

Where W_LDH−CuS_ is the mass of LDH-CuS added in the loading process, W_Fed_ is the initial total mass of fed SOR and W_Non−encapsulated_ is the SOR mass in the supernatant after centrifugation.

### NIR-responsive SOR release

1 mL of SOR@LDH-CuS/P hydrogel was placed into 10 mL tube with 10 mL of PBS added. The release of SOR from SOR@LDH-CuS/P hydrogel was determined by UV-vis spectroscopy. The SOR@LDH-CuS/P hydrogel was exposed to 1064 nm laser (0.5 W cm^− 2^) for 2 min with an interval of 2 h, which was repeated three times. For comparison, the SOR@LDH-CuS/P hydrogel without NIR irradiation was set as a control.

### The photothermal effect of LDH-CuS and SOR@LDH-CuS/P hydrogel

2 mL LDH-CuS suspension or SOR@LDH-CuS/P hydrogel (concentration of LDH-CuS: 100 µg mL^− 1^) were put into transparent quartz vial, respectively, followed by 1064 nm laser irradiation for a duration of 600 s at different power densities (0.05, 0.10, 0.20, 0.25 and 0.50 W cm^− 2^). The temperature change of each group was recorded by thermal infrared imaging camera (Fluke Ti450, USA) every 30 s for 10 min.

### Rheological characterization

Rheological experiments were carried out on the Thermo Mars40 rheometer to determine the injectability, gelation temperature and gelation time. The relationship between shearing rate and shearing stress was investigated to evaluate the injectability of SOR@LDH-CuS/P hydrogel at an angular frequency of 1 Hz, a strain of 1% and 25 ºC. The gelation temperature of SOR@LDH-CuS/P hydrogel was then detected from 25 to 40 °C (1 °C min^− 1^) at 1 Pa (stress) and 1 Hz (frequency). The time sweep rheological analysis of SOR@LDH-CuS/P hydrogel was performed at 25 °C and 1% of strain with a constant frequency of 1 rad s^− 1^.

### Mechanical measurement

The compress stress–strain measurements of SOR@LDH-CuS/P hydrogel and PNIPAAm were performed on a universal mechanical testing machine (MTS System Corporation, CMT6103). Briefly, the compressive tests of SOR@LDH-CuS/P hydrogel (13 mm in diameter and 7 mm in thickness) were performed at the speed of 1 mm min^− 1^.

### Ex vivo blood vessel squeezing and occlusion test.

All SD rattus norvegicus were purchased from Beijing Vital River Laboratory Animal Technology Co., Ltd. Two aorta vessels were stripped out from SD rattus norvegicus and rinsed by PBS. As for the experimental apparatus, DMEM was used to simulate blood with a flow rate set to 72 mL h^− 1^. The middle of aorta vessel was immersed into SOR@LDH-CuS/P hydrogel solution, and one tail end was connected to a controlled injection pump.

### Cell culture

Three types of cells (HepG2, Hela, 4T1) were purchased from the Institute of Basic Medical Sciences Chinese Academy of Medical Sciences (Beijing, China) and cultured in DMEM with 10% FBS and 1% penicillin/streptomycin at 37 °C under an atmosphere of 5% CO_2_.

### In vitro biosafety and cytotoxicity evaluation.

The standard MTT assay was utilized to evaluate the biocompatibility of LDH-CuS/P (LDH-CuS/PNIPAAm) hydrogel. Briefly, HepG2, Hela and 4T1 cells (1 × 10^4^ cells/well) were seeded into 96-well culture plates for 24 h incubation at 37 °C with 5% CO_2_, respectively. Then, 100 µL LDH-CuS/P hydrogel with different concentrations of LDH-CuS (10, 20, 50, 100, and 200 µg mL^− 1^) were added into 96-well plates for another 24 h incubation, respectively. After removing the previous medium, all treated cells were washed three times with PBS and then cultured with fresh DMEM medium containing MTT (500 µg mL^− 1^) for another 4 h. Finally, the cell viability was measured by microplate reader (at 490 nm). Subsequently, HepG2 cells were used to evaluate the cytotoxicity of SOR@LDH-CuS/P hydrogel. HepG2 cells were treated with 100 µL of PBS, NIR (0.5 W cm^− 2^, 6 min), LDH-CuS/P (concentration of LDH-CuS: 100 µg mL^− 1^), LDH-CuS/P + NIR (concentration of LDH-CuS: 100 µg mL^− 1^, 0.5 W cm^− 2^, 6 min), SOR@LDH-CuS/P (concentration of LDH-CuS: 100 µg mL^− 1^, LDH-CuS: SOR = 1: 2), and SOR@LDH-CuS/P + NIR (concentration of LDH-CuS: 100 µg mL^− 1^, LDH-CuS: SOR = 1: 2, 0.5 W cm^− 2^, 6 min) for 24 h, respectively. The NIR irradiation was performed at 12 h post-administration and divided into 3 times, each of two-minutes duration with an interval of 10 min. After that, HepG2 cells were washed three times with PBS and then cultured with 200 µL fresh DMEM medium containing MTT (500 µg mL^− 1^) for another 4 h. The cell viability was finally measured by microplate reader.

### Calcein-AM/PI staining

Calcein-AM/PI assay was utilized to visualize the therapeutic effect. Briefly, HepG2 cells (1 × 10^5^ cells/well) were inoculated into 6-well plates for 24 h incubation at 37 °C with 5% CO_2_, followed by treatment with 100 µL of PBS, NIR (0.5 W cm^− 2^, 6 min), LDH-CuS/P (concentration of LDH-CuS: 100 µg mL^− 1^), LDH-CuS/P + NIR (concentration of LDH-CuS: 100 µg mL^− 1^, 0.5 W cm^− 2^, 6 min), SOR@LDH-CuS/P (concentration of LDH-CuS: 100 µg mL^− 1^, LDH-CuS: SOR = 1: 2) and SOR@LDH-CuS/P + NIR (concentration of LDH-CuS: 100 µg mL^− 1^, LDH-CuS: SOR = 1: 2, 0.5 W cm^− 2^, 6 min) for 24 h, respectively. The NIR irradiation was performed at 12 h post-administration and divided into 3 times, each of two-minutes duration with an interval of 10 min. Subsequently, fresh DMEM containing Calcein-AM (2 mL, 5 µg mL^− 1^) and PI (2 mL, 10 µg mL^− 1^) were added into 6-well plates for 30 min incubation. All treated cells were washed with PBS three times and the corresponding fluorescence images were captured by a digital microscope (Leica).

### Apoptosis and necrosis assay

HepG2 cells were seeded into 6-well culture plates (1 × 10^5^ cells per well) at 37 °C for 24 h. Then, the previous medium was replaced by fresh one with 100 µL of PBS, NIR, LDH-CuS/P, LDH-CuS/P + NIR, SOR@LDH-CuS/P, and SOR@LDH-CuS/P + NIR for 24 h incubation, respectively. The concentration was consistent with the Calcein-AM/PI staining experiment. After co-culture with Annexin V-FITC/PI for 30 min, all treated cells were washed with PBS three times and then detected by flow cytometer.

### Animal experiments

All animals (Female Balb/c-nude mice, aged 3–5 weeks, 15–17 g body weight) were purchased from Beijing Vital River Laboratory Animal Technology Co., Ltd. All experiments were approved by the China-Japan Friendship Hospital Animal Research Center and complied with the guidelines of the Ethics Committee of China-Japan Friendship Hospital. To establish tumor model, HepG2 cells dispersed in 0.1 mL of PBS (1 × 10^7^ cells) were subcutaneously transplanted into the right hind legs of Balb/c-nude mice. The mean tumor was allowed to be 80 mm^3^ before all experiments began.

### In vivo antitumor therapy.

The Balb/c-nude mice bearing HepG2 tumors were randomly divided into six groups (*n* = 6): (1) control, (2) LDH-CuS/P (200 µL, NIPAAm: 20w%, LDH-CuS: 1 mg kg^− 1^), (3) LDH-CuS/P + NIR (200 µL, NIPAAm: 20w%, LDH-CuS: 1 mg kg^− 1^, 0.5 W cm^− 2^, 6 min), (4) SOR (200 µL, 2 mg kg^− 1^), (5) SOR@LDH-CuS/P (200 µL, NIPAAm: 20w%, SOR: 2 mg kg^− 1^, LDH-CuS: 1 mg kg^− 1^), (6) SOR@LDH-CuS/P + NIR (200 µL, NIPAAm: 20w%, SOR: 2 mg kg^− 1^, LDH-CuS: 1 mg kg^− 1^). The NIR irradiation was performed at 12 h post-injection and divided into 3 times, each of two-minutes duration with an interval of 10 min. The dosage of SOR was referred to previous reports. The body weight and tumor size were recorded every 2 days for 16 days.

### In vivo vascular occlusion test on blood vessels.

200 µL of SOR@LDH-CuS/P (NIPAAm: 20w%, LDH-CuS: 1 mg kg^− 1^; SOR: 2 mg kg^− 1^) was intratumorally injected into mice. Power doppler images were captured at pre-, post- and 7 days post-treatment with SOR@LDH-CuS/P by Visual Sonic Vevo + 3100.

### Intratumoral vascular occlusion

To evaluate the occluded blood supply caused by the extravascular gelation shrinkage, in vivo perfusion assay was carried out using FITC-labeled dextran *via* fluorescence imaging technique. Mice were randomly separated into two groups (*n* = 3): PBS (control: 200 µL) and SOR@LDH-CuS/P (treated: 200 µL, NIPAAm: 20w%, LDH-CuS: 1 mg kg^− 1^; SOR: 2 mg kg^− 1^). FITC-labeled dextran was intravenously injected into mice. The fluorescence images of all treated mice were captured by the in vivo imaging system (IVIS, PerkinElmer, USA) after 10 min post-injection.

### Biodistribution and retention studies

An in vivo fluorescence imaging system was used to investigate the localized retention ability of the hydrogel over the whole body. Mice were randomly separated into two groups (*n* = 3): SOR@LDH-CuS/P (200 µL, NIPAAm: 20w%, LDH-CuS: 1 mg kg^− 1^; SOR: 2 mg kg^− 1^) and SOR@LDH-CuS (200 µL, LDH-CuS: 1 mg kg^− 1^; SOR: 2 mg kg^− 1^), respectively. Then, the fluorescence images of all treated mice were captured by an IVIS imaging system (Perkin Elmer, USA) at five time points, 0, 1st, 3rd, 7th, and 14th days. Afterwards, the mice were sacrificed on the 14th day post-injection, and the fluorescence intensity of major organs (heart, liver, spleen, lung, kidney, tumor) was also recorded.

### Pathological investigation

All the mice in six groups were sacrificed after 16 days of treatment. Then, the tumors and major organs of each group were collected and preserved in 4% paraformaldehyde solution for histology analysis. All tissue slices were observed with a Leica digital microscope after stained with H&E and TUNEL.

### Immunofluorescence staining

We also investigated the variation of blood vessels of mice after different treatments. FITC-labeled CD34 and Cy 3-labeled CD31 immunofluorescence staining assays were performed on tumor slices. The corresponding fluorescence images were then captured by a Leica digital microscope.

### Histology examination

Mice in two groups (PBS and SOR@LDH-CuS/P) were sacrificed after 16 days of treatment. Then, heart, liver, spleen, lungs and kidneys were collected and preserved in 4% paraformaldehyde solution for histology analysis. All tissue slices were observed with a Leica digital microscope after stained with H&E.

### Statistical analysis.

Data were presented as mean value ± standard deviation (SD). The statistical significances between the two groups were analyzed by unpaired Student’s t test: *p < 0.05, **p < 0.01, ***p < 0.001.

### Electronic supplementary material

Below is the link to the electronic supplementary material.

Supplementary Material 1

## Data Availability

No datasets were generated or analysed during the current study.
