# Supplementary material for: A MgAl-LDH-CuS nanosheet-based thermo-responsive composite hydrogel with nir-responsive angiogenesis inhibitor releasing capability for multimode starvation therapy
Source: J Nanobiotechnology. 2024 Mar 23;22:127. doi: 10.1186/s12951-024-02384-w (PMC10960490; doi:10.1186/s12951-024-02384-w)
Supplement: Supplementary file 1 — Supplementary Material 1 [file 12951_2024_2384_MOESM1_ESM.docx]

***Supporting information***

**A MgAl-LDH-CuS Nanosheet-based Thermo-Responsive Composite Hydrogel with NIR-Responsive** **Angiogenesis Inhibitor Releasing Capability for Multimode Starvation Therapy**

Xueyan Liu,^†^ Tingting Hu,^†^ Yijiang Jia, Shuqing Yang, Yu Yang, Zhuolin Cui, Tao Wang, Ruizheng Liang,* Chaoliang Tan,* and Yuji Wang*

^†^These authors contributed equally: Xueyan Liu, Tingting Hu.

X. Liu, Y. Jia and Prof. Y. Wang

School of Pharmaceutical Sciences of Capital Medical University, No.10 Xitoutiao, You An Men, Beijing 100069, P. R. China

Laboratory for Clinical Medicine, Engineering Research Center of Endogenous Prophylactic of Ministry of Education of China, Beijing Laboratory of Biomedical Materials, Beijing 100069, P. R. China

E-mail: wangyuji@ccmu.edu.cn (Y. Wang)

X. Liu, S. Yang, Y. Yang, Z. Cui, T. Wang and Prof. R. Liang

State Key Laboratory of Chemical Resource Engineering, Beijing Advanced Innovation Center for Soft Matter Science and Engineering, Beijing University of Chemical Technology, Beijing 100029, P. R. China

E-mail: liangrz@mail.buct.edu.cn (R. Liang)

Prof. R. Liang

Quzhou Institute for Innovation in Resource Chemical Engineering, Quzhou 324000, P. R. China

T. Hu and Prof. C. Tan

Department Electrical and Electronic Engineering, The University of Hong Kong, Pokfulam Road, Hong Kong SAR 999077, P. R. China

E-mail: cltan@hku.hk (C. Tan)

**Table S1** The metal element ratios of MgAl-LDH and LDH-CuS nanosheets detected by ICP-MS.

| n (mol) | Mg | Al | Cu |
| --- | --- | --- | --- |
| MgAl-LDH | 2.38 | 1 | \ |
| LDH-CuS | 1.93 | 0.83 | 0.51 |

**Fig. S1** Size distribution of LDH, LDH-CuS and SOR@LDH-CuS nanosheets.


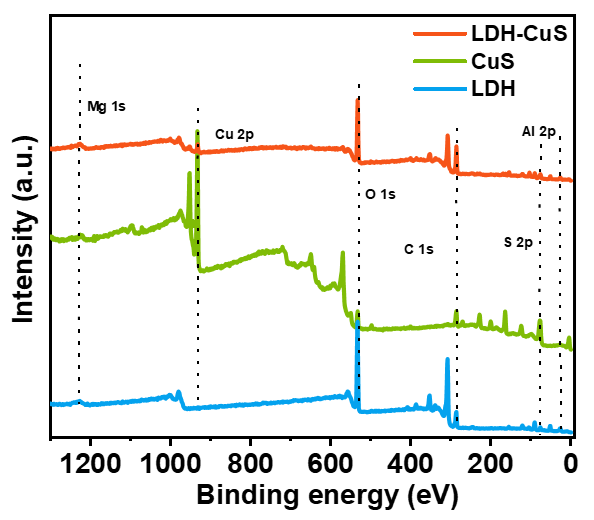


**Fig. S2** XPS spectra of CuS nanodots, MgAl-LDH and LDH-CuS nanosheets.


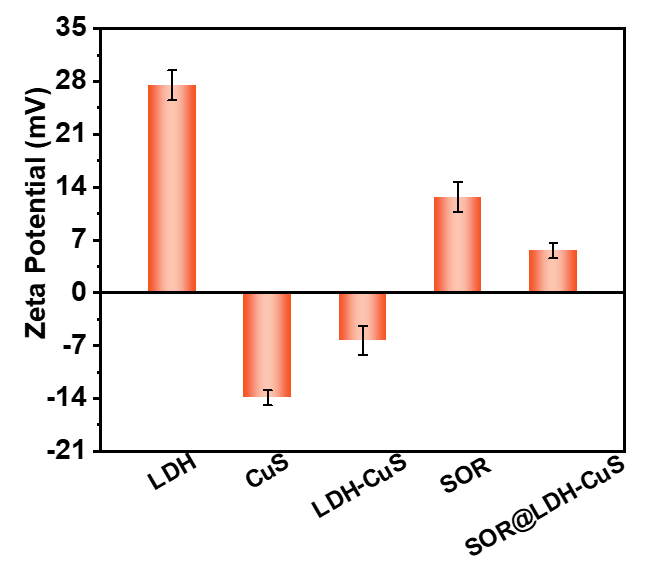


**Fig. S3** Zeta potentials of MgAl-LDH nanosheets, CuS nanodots, LDH-CuS nanosheets, SOR, and SOR@LDH-CuS nanosheets.

**
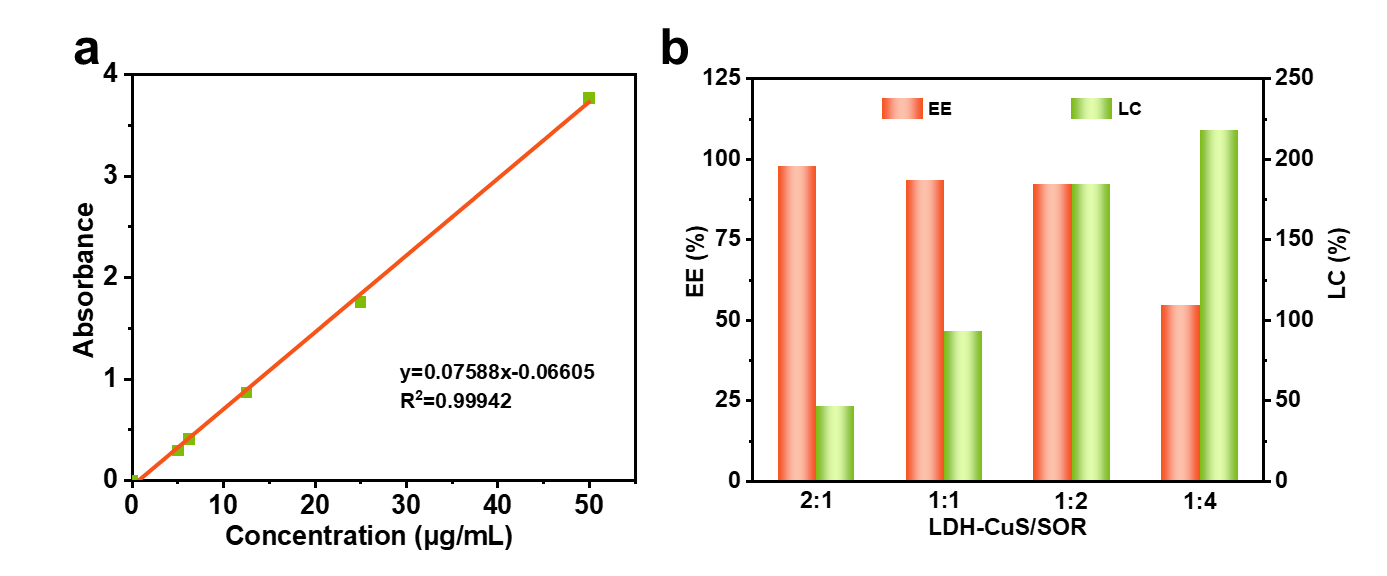
**

**Fig. S4** (a) Equation of linear regression of SOR detected by UV-vis absorption spectroscopy. (b) Load capacity and entrapment efficiency of SOR in LDH-CuS nanosheets.


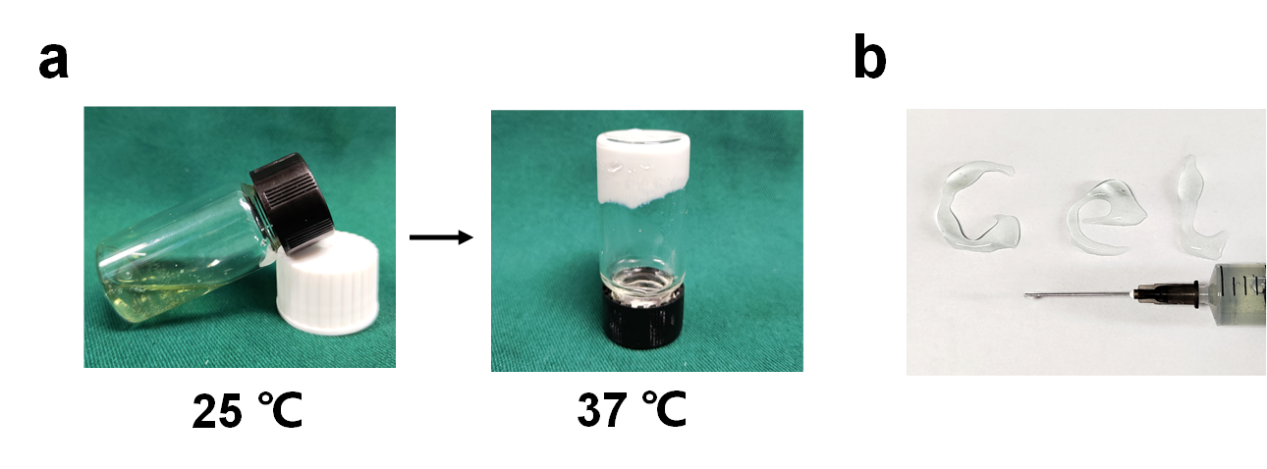


**Fig. S5** (a) Digital photographs of SOR@LDH-CuS-100/P at different temperatures. (b) Digital photograph of the green SOR@LDH-CuS-100/P hydrogel injected *via* a fine needle.


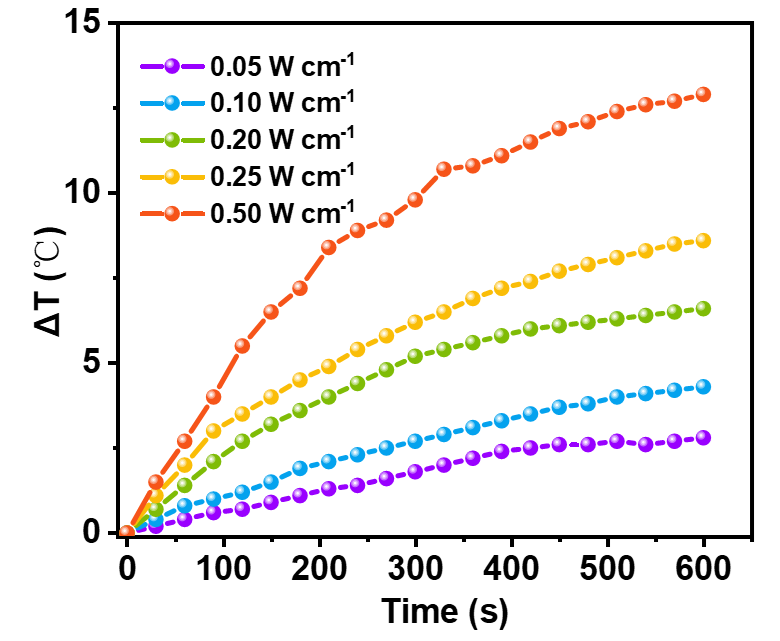


**Fig. S6** Temperature profiles of LDH-CuS (100 μg mL^−1^) under 1064 nm laser irradiation at various power densities (0.05, 0.10, 0.20, 0.25, and 0.5 W cm^−2^).


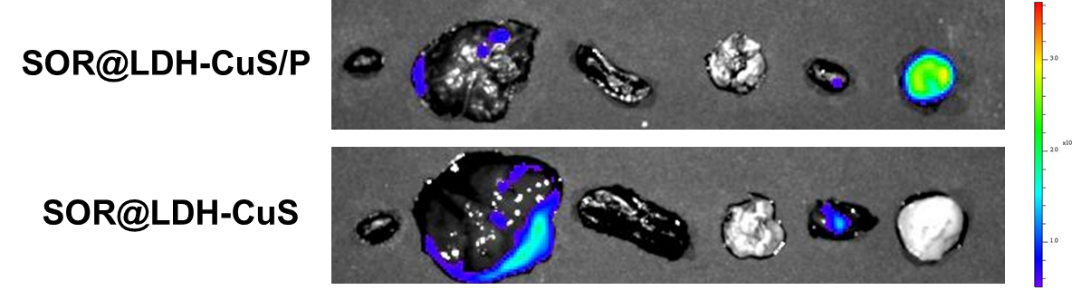


**Fig. S7** *In vivo* biodistribution and retention of SOR@LDH-CuS/P and SOR@LDH-CuS analyzed by fluorescence imaging. Images of major organs of mice after injection of SOR@LDH-CuS/P and SOR@LDH-CuS at 14^th^ day.


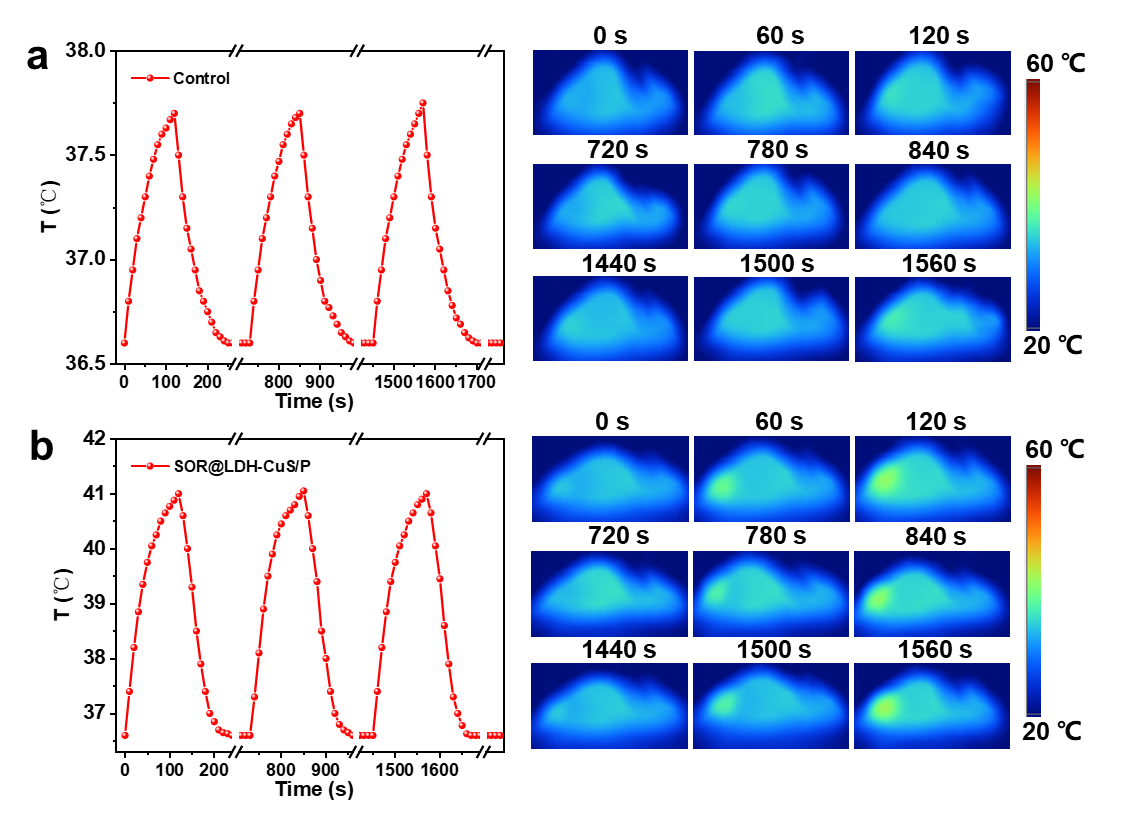


**Fig. S8** Temperature variation and photothermal imaging of tumors of mice treated with (a) 1064 nm laser and (b) SOR@LDH-CuS/P + 1064 nm laser (0.5 W cm^−2^, 10 min).

**Fig. S9** Tumor growth inhibition rates of mice after different treatments.

**Fig. S10** Body weight changes of mice with different treatments.


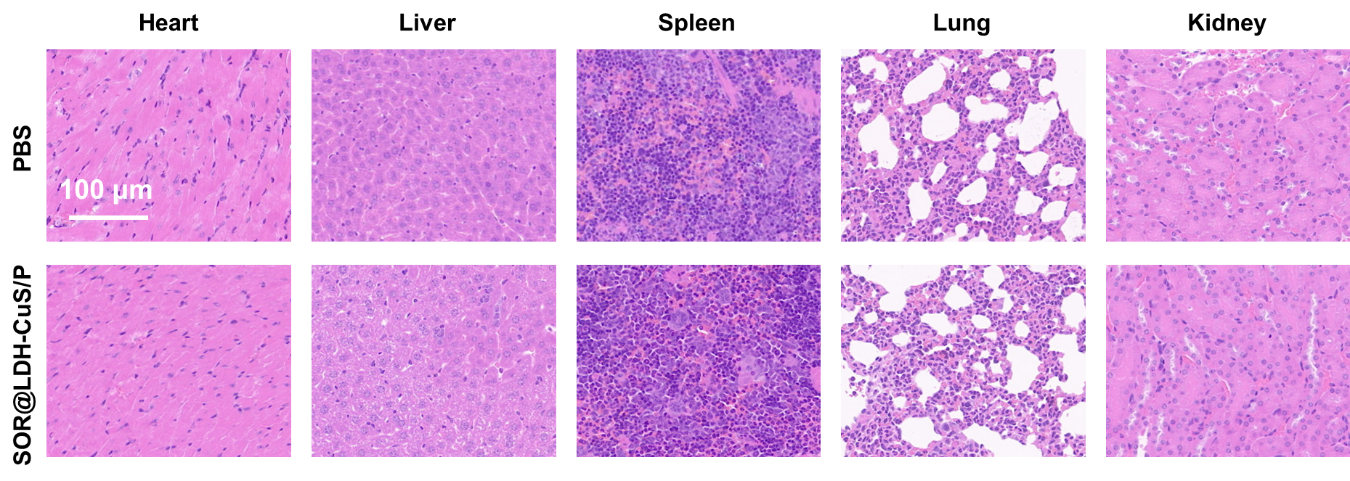


**Fig. S11** H&E staining images of major organs collected on day 16 from mice treated with PBS and SOR@LDH-CuS/P, respectively.


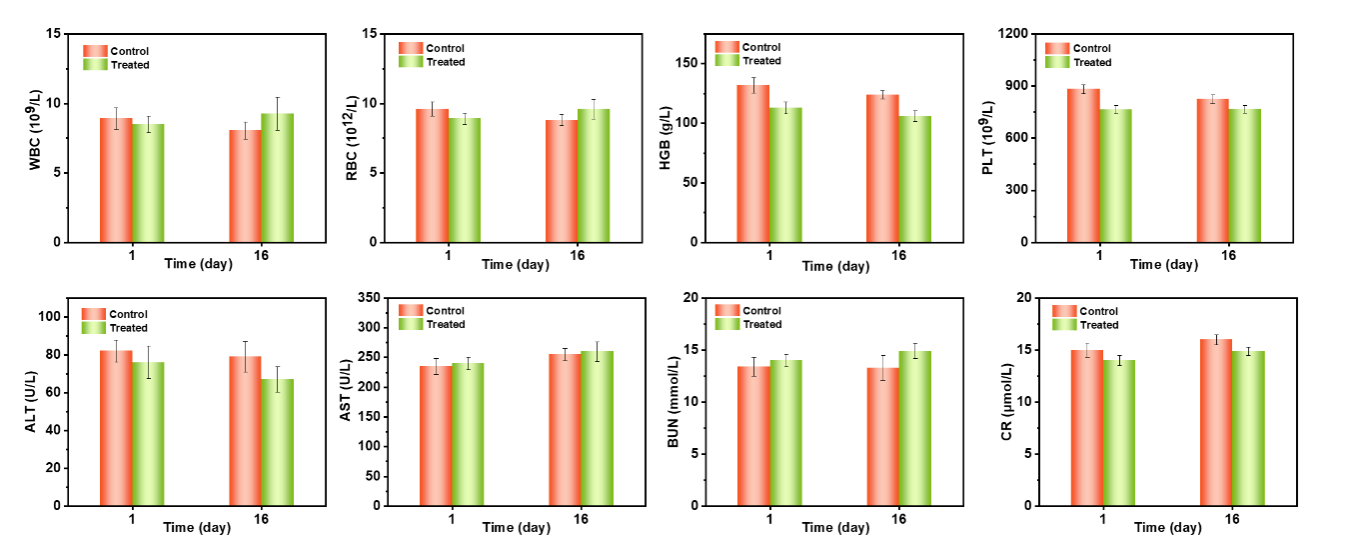


**Fig. S12** Blood cell counts and kidney/liver function markers of mice after injection of PBS (control) and SOR@LDH-CuS/P (treated) at day 1 and day 16, respectively.

**Fig. S13** Survival rate of mice with different treatments: 1) PBS + NIR, 2) LDH-CuS/P, 3) LDH-CuS/P + NIR, 4) SOR, 5) SOR@LDH-CuS/P, 6) SOR@LDH-CuS/P + NIR.
